# JP1 normalizes tumor vasculature to suppress metastasis and facilitate drug delivery by inhibiting IL-8

**DOI:** 10.1172/jci.insight.161675

**Published:** 2023-06-22

**Authors:** Jiahua Cui, Zhen Che, Lu Zou, Dongyin Chen, Zhan Xie, Kun Ding, Huning Jiang, Aiping Li, Jianwei Zhou, Yongqian Shu

**Affiliations:** 1 Department of Oncology, The First Affiliated Hospital of Nanjing Medical University, Nanjing, China.; 2 Department of Epidemiology, School of Public Health, Nantong University, Nantong, China.; 3 Department of Molecular Cell Biology & Toxicology, Center for Global Health, School of Public Health,; 4 Jiangsu Key Lab of Cancer Biomarkers, Prevention and Treatment, Collaborative Innovation Center for Cancer Medicine, and; 5 Department of Medicinal Chemistry, School of Pharmacy, Nanjing Medical University, Nanjing, China.; 6 Department of Ophthalmology, The First Affiliated Hospital of Nanjing Medical University, Nanjing, China.; 7 Department of Oncology, Sir Run Run Hospital, and; 8 Jiangsu Key Lab of Cancer Biomarkers, Prevention and Treatment, Collaborative Innovation Center for Cancer Personalized Medicine, Nanjing Medical University, Nanjing, China.

**Keywords:** Angiogenesis, Oncology, Cancer, Drug therapy

## Abstract

Tumor vascular normalization prevents tumor cells from breaking through the basement membrane and entering the vasculature, thereby inhibiting metastasis initiation. In this study, we report that the antitumor peptide JP1 regulated mitochondrial metabolic reprogramming through AMPK/FOXO3a/UQCRC2 signaling, which improved the tumor microenvironment hypoxia. The oxygen-rich tumor microenvironment inhibited the secretion of IL-8 by tumor cells, thereby promoting tumor vascular normalization. The normalized vasculature resulted in mature and regular blood vessels, which made the tumor microenvironment form a benign feedback loop consisting of vascular normalization, sufficient perfusion, and an oxygen-rich microenvironment, prevented tumor cells from entering the vasculature, and inhibited metastasis initiation. Moreover, the combined therapy of JP1 and paclitaxel maintained a certain vascular density in the tumor and promoted tumor vascular normalization, increasing the delivery of oxygen and drugs and enhancing the antitumor effect. Collectively, our work highlights the antitumor peptide JP1 as an inhibitor of metastasis initiation and its mechanism of action.

## Graphical abstract

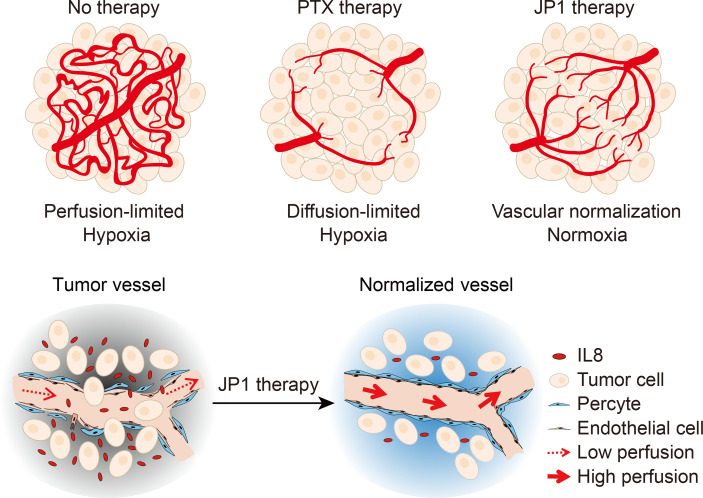

## Introduction

Recently, emerging tumor diagnostic and therapeutic technologies have remarkably improved the cure rate of primary tumors, and some have even completely inhibited tumor growth at certain stages (1, 2). However, tumor metastasis is the main cause of death in tumor patients (3). Once metastases are established, existing therapies generally fail to provide durable responses. Since the therapeutic strategies for tumor metastasis are limited, preventing metastasis initiation may be the next breakthrough for tumor therapy (4, 5). The precolonization phase of metastasis is a sophisticated multistep process in which tumor cells shed from the primary tumor, penetrate the basement membrane, enter the vasculature, take up residence in the target organ, and eventually form metastases (6). Tumor cells breaking through the basement membrane and entering the vasculature are the core of initiating tumor metastasis (7). The immature and unstable vasculature of the tumor microenvironment provides conditions for primary tumor cells to enter the vasculature and metastasize to distant organs (8, 9). Therefore, remodeling the disordered vasculature and correcting it into a mature and stable vasculature may effectively inhibit tumor metastasis.

Tumor angiogenesis is a regenerative process that is highly affected by tumor cells and facilitates tumor growth and metastasis (10). Tumor cells secrete a large number of cytokines to interact with perivascular cells and transform them into fibroblasts, thus making those cells lose their role in protecting blood vessels (11, 12). Moreover, tumor cells release pseudo-endothelial signals to stimulate endothelial cell migration, which leads to a discontinuous endothelial lining and defective basement membrane (13). All these features are signs of immature and dysfunctional vessels. Consequently, vascular permeability remains high, which provides a means for tumor cells to invade blood vessels. Tumor vascular normalization results in a resting state of endothelial cells surrounded by mature pericytes. Endothelial cells and pericytes are separated by a basement membrane that is filled with various adhesion proteins to maintain vascular stability (14). Tumor vascular normalization suppresses metastasis by reducing the invasion of tumor cells into the vasculature (15, 16). Moreover, the regular and mature blood vessels in the tumor microenvironment promote blood perfusion and relieve hypoxia and acidosis (17).

IL-8, a known chemotactic cytokine, has been reported to be highly expressed in a variety of tumors (18, 19). It promotes malignant proliferation and metastasis and plays a key regulatory role in the tumor microenvironment (20, 21). IL-8 also aggravates tumorigenicity by mediating the crosstalk between endothelial colony-forming cells and triple-negative breast cancer (22). The hepatitis B virus protein HBx induces high IL-8 production through MEK/ERK signal activation, leading to enhanced endothelial permeability to facilitate tumor vascular invasion (23). More importantly, the tumor microenvironment hypoxia is induced by IL-8–mediated vascular immaturity, which in turn stimulates tumor cells to secrete IL-8 (24). These cause a malignant feedback loop consisting of tumor microenvironment hypoxia, IL-8 secretion, and vascular disturbances. Therefore, the inhibition of IL-8 may be an effective therapy to correct the disordered vasculature in the tumor microenvironment.

The JWA gene, which encodes ADP ribosylation factor–like GTPase 6–interacting protein 5 (ARL6IP5, hereafter JWA), was initially cloned from retinoic acid–induced HBE cell differentiation models (25). Studies have shown that JWA inhibits the malignant phenotypes of melanoma, gastric cancer, breast cancer, etc. (26–28). JWA promotes mitochondrial metabolic reprogramming by regulating AMPK/FOXO3a/UQCRC2 signaling to improve the tumor microenvironment hypoxia, thereby inhibiting tumor metastasis (29). The antitumor peptide JP1 was designed as a pre-phosphorylated 7–amino acid fragment based on the functional sequence of the JWA protein and with an RGD linker to target integrin αvβ3. JP1 effectively restricts melanoma metastasis through MEK1/-2/NEDD4L/SP1/integrin αvβ3 signaling (30). However, our understanding of how JP1 inhibits tumor metastasis remains limited. In this study, we show that JP1 inhibits IL-8 expression though improving the tumor microenvironment hypoxia, which promotes the normalization of tumor vasculature. Tumor vascular normalization inhibits tumor metastasis and enhances the delivery and uniform intratumoral distribution of paclitaxel (PTX).

## Results

### JP1 inhibits metastasis by reducing tumor cells in the vasculature.

**Figure 1:**
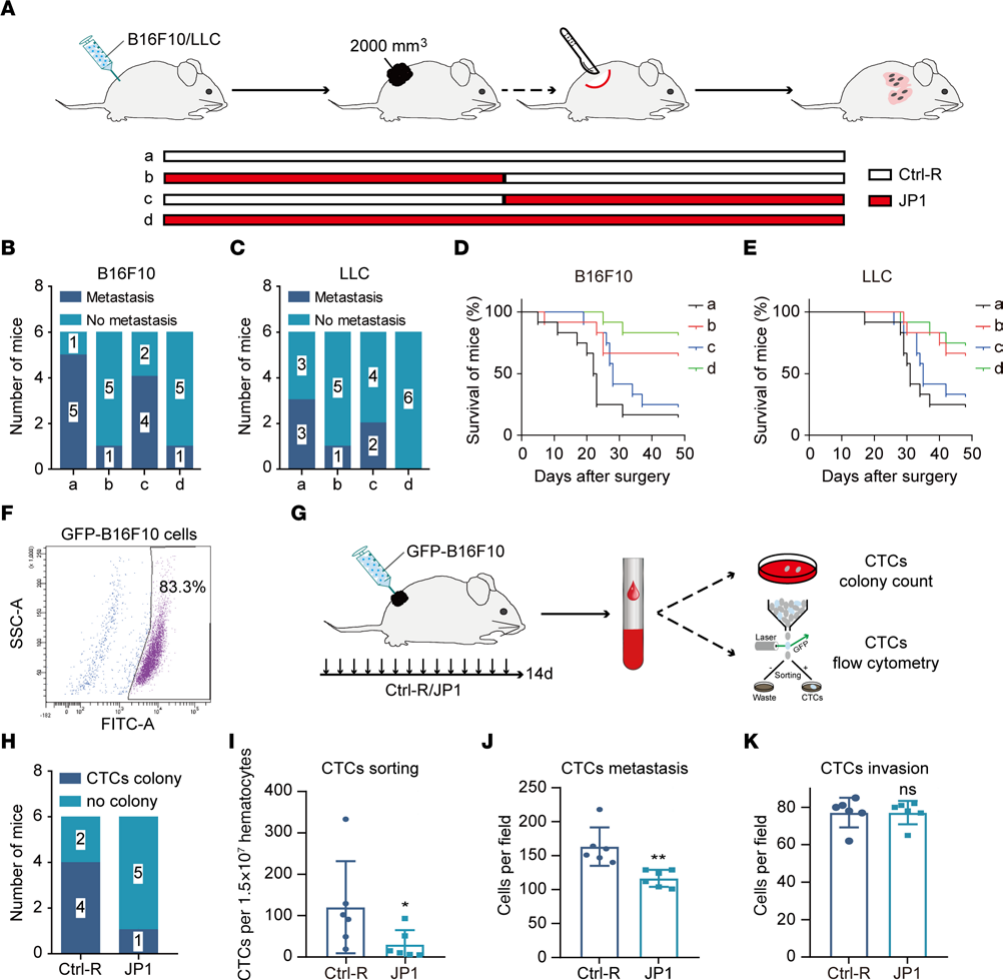
JP1 inhibits metastasis by reducing tumor cells in the vasculature. (**A**) Schematic representation of the B16F10 and LLC cell allografts and active metastasis 2-stage models for Ctrl-R (50 mg/kg/day) or JP1 (50 mg/kg/day) treatment. (**B** and **C**) Quantitation of the mice that developed lung metastases after surgical removal of the primary B16F10 (**B**) or LLC tumors (**C**) with indicated treatments (*n* = 6). (**D** and **E**) Kaplan-Meier survival analysis of mice with indicated B16F10 (**D**) and LLC (**E**) tumors (*n* = 12). (**F**) FACS analysis of GFP^+^ B16F10 cells after transfection with GFP-specific plasmids. (**G**) Schematic of CTC analysis by CTC colony count and flow cytometry. (**H**) Quantitation of the colony formation for CTCs after Ctrl-R or JP1 treatment (*n* = 6). (**I**) Quantitation of CTCs per 1.5 million hematocytes after Ctrl-R or JP1 treatment (*n* = 6). (**J** and **K**) CTCs derived from 6 mice were analyzed in the migration and invasion assays. Quantitation of migrating cells (**J**) and invading cells (**K**) per 30,000 cells. *P < 0.05, **P < 0.01 by unpaired, 2-tailed Student’s *t* test (**I**–**K**).

To identify the role of JP1 in metastasis initiation, we established active metastasis models by surgical removal of the primary tumor (Figure 1A). Following primary tumor resection, the mice developed multiorgan metastases, and the most common was lung metastases. Subsequently, we performed lung histological staining to determine the incidence of lung metastasis and found that 5 out of 6 mice in the control (Ctrl-R) group showed lung metastasis, whereas, only 1 out of 6 mice had lung metastasis in the JP1 group in the melanoma model. Importantly, lung metastasis only occurred in 1 out of 6 mice even if the JP1 treatment was stopped after primary tumor resection, whereas 4 out of 6 mice showed lung metastasis in the group given JP1 treatment after the removal of the primary tumor (Figure 1B and Supplemental Figure 1, A–C; supplemental material available online with this article; https://doi.org/10.1172/jci.insight.161675DS1). This observation was further validated in a lung cancer active metastasis model (Figure 1C and Supplemental Figure 1, D–F). Moreover, parallel survival models were monitored. It was found that, compared with the control, JP1 treatment given before primary tumor resection effectively prolonged mouse survival in the melanoma and lung cancer models. However, JP1 treatment given after primary tumor resection had little effect on prolonging mouse survival (Figure 1, D and E). These data support the notion that JP1 inhibits tumor metastasis.

To further validate this hypothesis, we used GFP-labeled B16F10 cells to construct a melanoma-bearing model and analyzed circulating tumor cells (CTCs) by cell cloning and FACS (Figure 1, F and G). The cell cloning analysis showed that 4 out of 6 CTCs extracted from mice in the Ctrl-R group formed clonal clusters in vitro, while only 1 out of 6 in the JP1 group did so (Figure 1H and Supplemental Figure 2A). In addition, FACS analysis showed that tumor cells from primary tumors entering the circulatory system significantly decreased after JP1 treatment (Figure 1I and Supplemental Figure 2B). We then carried out CTC migration and invasion assays and found that the migratory ability of CTCs after JP1 treatment was weakened (Figure 1J and Supplemental Figure 2C), but the invasiveness remained unchanged (Figure 1K and Supplemental Figure 2D). These results indicate that JP1 inhibits metastasis initiation by reducing the number of tumor cells entering the vasculature, but has no discernible effect on the invasiveness of tumor cells.

### JP1 promotes tumor vascular normalization.

**Figure 2:**
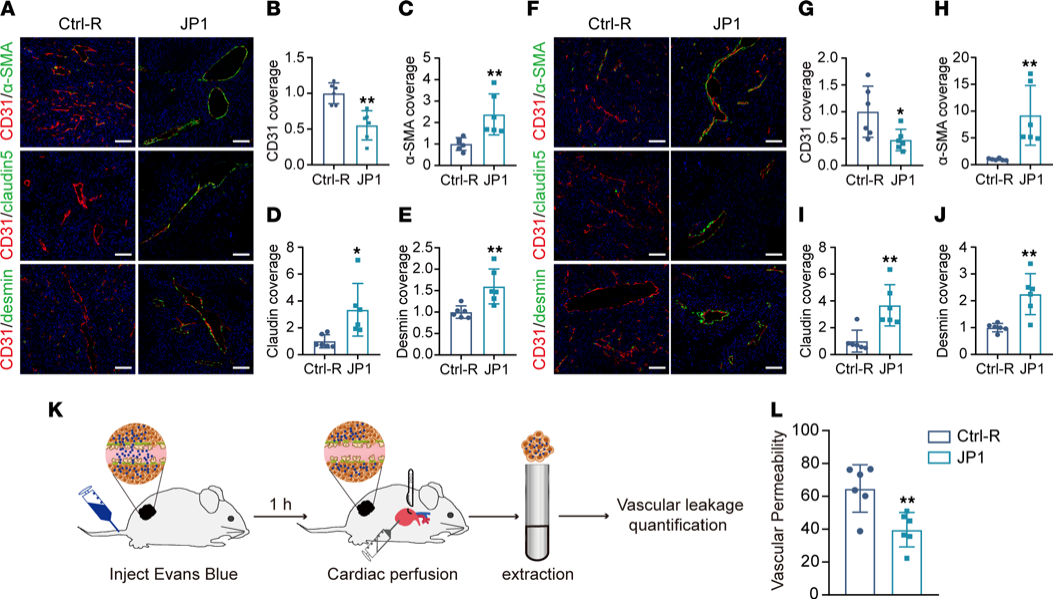
JP1 promotes tumor vascular normalization and reduces vascular permeability. (**A**) Representative fluorescence images of α-SMA (green), claudin 5 (green), desmin (green), CD31 (red), and DAPI nuclear staining of B16F10 tumor nodules treated with Ctrl-R or JP1. (**B**–**E**) The CD31 coverage (**B**), α-SMA coverage (**C**), claudin 5 coverage (**D**), and desmin coverage (**E**) in the interstitial microenvironment of B16F10 tumor nodules (*n* = 6). Scale bars: 100 μm. (**F**) Representative fluorescence images of α-SMA (green), claudin 5 (green), desmin (green), CD31 (red), and DAPI nuclear staining of LLC tumor nodules treated with Ctrl-R or JP1. (**G**–**J**) The CD31 coverage (**G**), α-SMA coverage (**H**), claudin 5 coverage (**I**), and desmin coverage (**J**) in the interstitial microenvironment of LLC tumor nodules (*n* = 6). Scale bars: 100 μm. (**K** and **L**) Schematic of vessel permeability assays (**K**) and quantitation of vascular permeability (**L**) after Ctrl-R or JP1 treatment (*n* = 6). *P < 0.05, **P < 0.01 by unpaired, 2-tailed Student’s *t* test (**B**–**D**, **E**, **G**–**J**, and **L**).

To investigate the function of JP1 that inhibits tumor cells from entering the vasculature, we assessed the morphology of tumor vessels extracted from melanoma and lung cancer samples. Immunofluorescent staining illustrated that the CD31 (marker of blood vessels) coverage was lower after JP1 treatment (Figure 2, A and B); however, the α-SMA (marker of mural cells), claudin 5 (marker of endothelial cells), and desmin (marker of pericytes) coverage was higher after JP1 treatment in melanoma (Figure 2, C–E). This observation was further demonstrated in lung cancer (Figure 2, F–J). These results showed that, compared with the control, the tumor showed vascular maturation phenotypes after JP1 treatment. Then, we carried out vessel permeability assays and revealed that, compared with Ctrl-R treatment, JP1 reduces vascular permeability (Figure 2, K and L). In short, JP1 restricted the entry of tumor cells into the vasculature by promoting tumor vascular normalization, thereby inhibiting tumor metastasis.

### JP1 promotes tumor vascular normalization by inhibiting IL-8.

**Figure 3:**
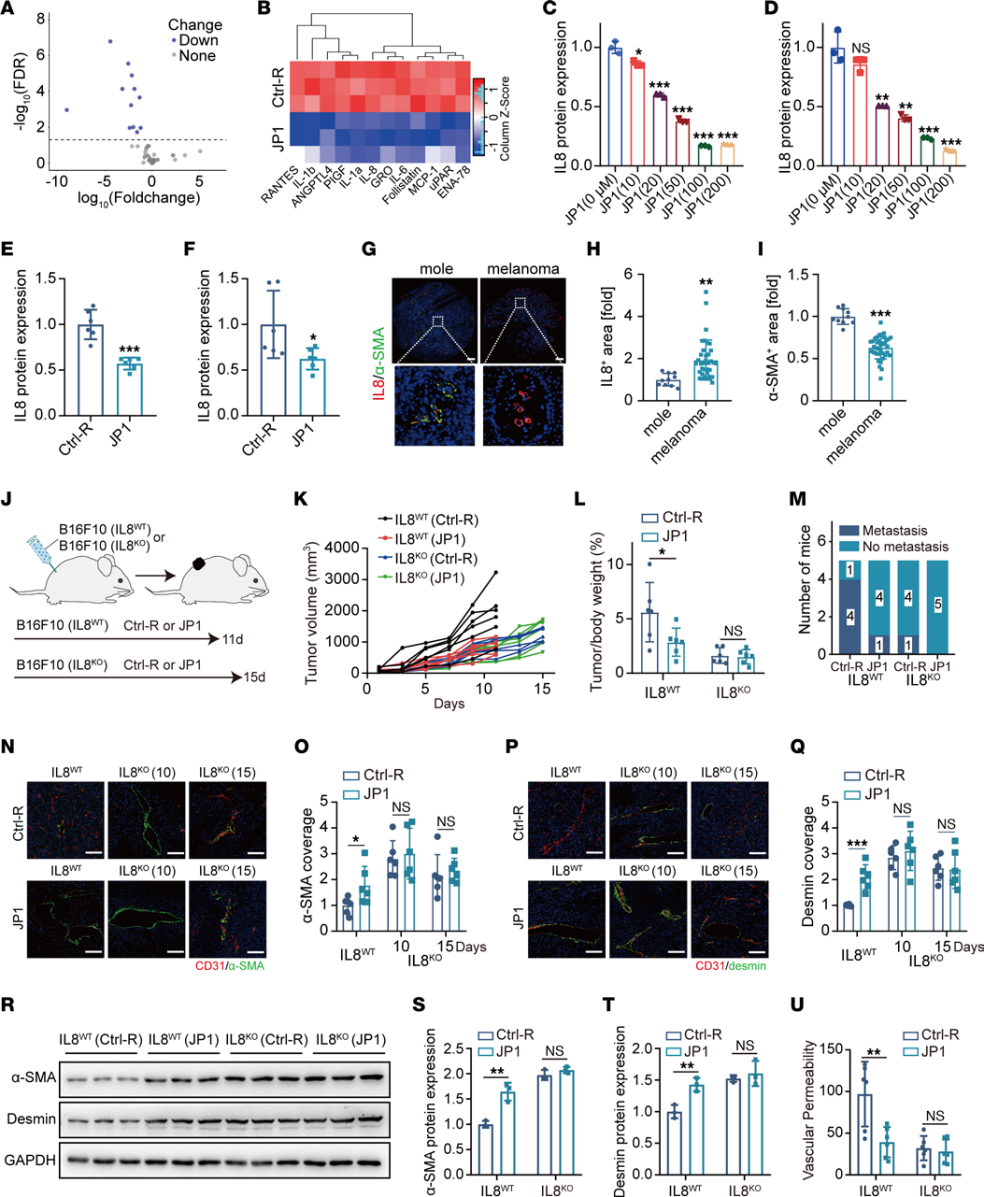
JP1 promotes tumor vascular normalization by inhibiting IL-8. (**A**) Volcano plot of 60 genes involved in tumor vascular normalization among Ctrl-R– or JP1-treated (50 μM for 48 hours) B16F10 cells (*n* = 3). (**B**) Heatmap of 12 significantly downregulated proteins after JP1 treatment (*n* = 3). (**C** and **D**) Quantitation of IL-8 protein after JP1 treatment with indicated concentrations for 48 hours in B16F10 (**C**) and LLC (**D**) cells (*n* = 3). (**E** and **F**) IL-8 protein extracted from B16F10 (**E**) and LLC (**F**) tumor nodules was assessed by Western blot; quantitation of IL-8 protein concentrations is shown (*n* = 6). (**G**) Representative immunofluorescent staining of α-SMA (green) and IL-8 (red) in human mole and melanoma. Scale bars: 200 μm. The dashed box is further magnified by 74×. (**H** and **I**) quantitation of IL-8^+^ area (**H**) and α-SMA^+^ area (**I**) between mole and melanoma. (**J**) Schematic representation of the B16F10 (IL8^WT^) and B16F10 (IL8^KO^) cell allografts for Ctrl-R (50 mg/kg/day) or JP1 (50 mg/kg/day) treatment (*n* = 6). (**K** and **L**) The tumor growth curves and tumor/body weight (%) in indicated groups. (**M**) Quantitation of the mice that developed lung metastases after surgical removal of the primary tumors with indicated treatments (*n* = 5). (**N** and **O**) Representative fluorescence images of α-SMA (green), CD31 (red), and DAPI nuclear staining in indicated groups (**N**) and quantitation of α-SMA coverage (**O**) (*n* = 6). Scale bars: 100 μm. (**P** and **Q**) Representative fluorescence images of desmin (green), CD31 (red), and DAPI nuclear staining in indicated groups (**P**) and quantitation of desmin coverage (**Q**) (*n* = 6). Scale bars: 100 μm. (**R**–**T**) The proteins extracted from B16F10 tumor nodules in indicated groups were assessed by Western blot (**R**); quantitation of α-SMA (**S**) and desmin (**T**) protein concentrations is shown (*n* = 3). (**U**) Quantitation of vascular permeability in indicated groups (*n* = 6). *P < 0.05; **P < 0.01; ***P < 0.001 by ordinary 1-way ANOVA (**C**, **D**, **L**, **O**, **Q**, and **S**–**U**) or unpaired, 2-tailed Student’s *t* test (**E**, **F**, **H**, and **I**).

In order to gain better molecular insight, we performed angiogenesis array analysis (Supplemental Table 2) and quantitative PCR (qPCR) assays to explore differentially regulated genes following JP1 treatment, which indicated that IL-8, IL-6, IL-1β, and heparin-binding EGF-like growth factor (HB-EGF) were the significantly downregulated genes (fold change ≥ 2 or ≤ 0.5 in both angiogenesis array analysis and qPCR assays) (Figure 3, A and B, and Supplemental Figure 3, A and B). The ELISA results further verified that IL-8 was the most downregulated gene (fold change < 0.5) after JP1 treatment (Supplemental Figure 3C). Western blot analysis elucidated that IL-8 was prominently decreased with increasing JP1 concentration (Figure 3, C and D, and Supplemental Figure 3D), a finding that we subsequently confirmed in mouse tumor samples (Figure 3, E and F, and Supplemental Figure 3E). The qPCR analysis also validated the in vivo results (Supplemental Figure 3, F and G). We further detected the expression of IL-8 in human melanocytic nevi and melanoma tissue microarray. As predicted, the expression of IL-8 in melanoma was significantly higher than melanocytic nevi (Figure 3, G and H). By contrast, mural cell coverage was lower in melanoma tissue (Figure 3I). These results demonstrated that IL-8, inhibited by JP1, was negatively correlated with vascular normalization.

For the purpose of exploring how IL-8 is regulated by JP1, we knocked out the *Il8* gene in B16F10 cells through CRISPR/Cas9 (Supplemental Figure 4A) and observed that JP1 promoted tumor vascular normalization in the absence of IL-8. We then used B16F10 cells with IL8^WT^ and IL8^KO^ to construct a melanoma-bearing model (Figure 3J). The results demonstrated that the growth rate of IL8^KO^ B16F10 cells was significantly lower than that of IL8^WT^ B16F10 cells. Moreover, compared with IL8^WT^ B16F10 cells, JP1 significantly inhibited melanoma growth, but had no obvious inhibitory effect on IL8^KO^ B16F10 cells (Figure 3, K and L, and Supplemental Figure 4, B and C). Subsequently, we created a melanoma active metastasis model by injecting IL8^WT^ or IL8^KO^ cells. After injecting IL8^WT^ cells, 4 out of 5 mice in the Ctrl-R group showed lung metastasis, whereas only 1 out of 5 mice was observed with lung metastasis in the JP1 group. After injecting IL8^KO^ cells, 1 out of 5 mice showed lung metastasis in the Ctrl-R group, and no mice were observed with lung metastasis in the JP1 group (Figure 3M and Supplemental Figure 4D). The morphological evaluation of vessels extracted from mouse tumor samples produced identical results. JP1 promoted tumor vascular normalization by increasing pericyte and endothelial cell coverage in IL8^WT^ B16F10; however, on the 10th and 15th days of tumor growth, the knockout of IL-8 significantly improved tumor vascular normalization, and JP1 treatment could not enhance the vascular normalization index (Figure 3, N–Q). This observation was further demonstrated by Western blot analysis (Figure 3, R–T). Vessel permeability assays confirmed that JP1 effectively reduces vascular permeability in IL8^WT^ B16F10; however, JP1 could not further reduce vascular permeability in IL8^KO^ B16F10 (Figure 3U). In summary, these data support the notion that JP1 promotes tumor vascular normalization by inhibiting IL-8.

### JP1 promotes oxidative phosphorylation and improves tumor microenvironment hypoxia.

**Figure 4:**
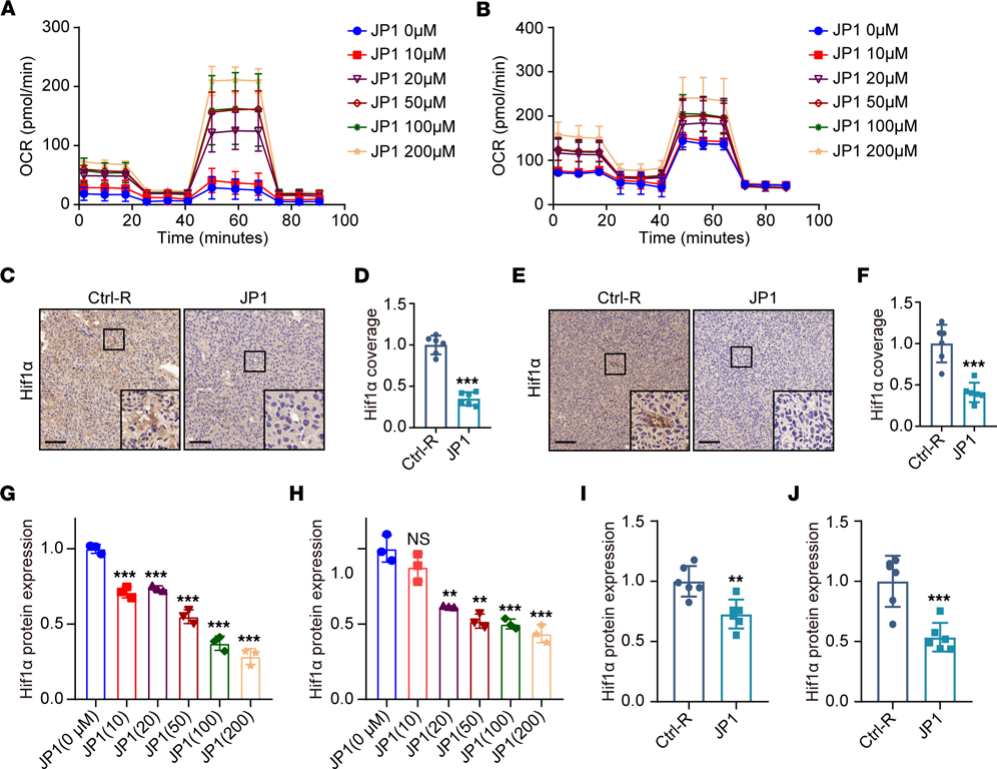
JP1 promotes tumor oxidative phosphorylation and improves tumor microenvironment hypoxia. (**A** and **B**) The oxidative phosphorylation level of B16F10 (**A**) and LLC (**B**) cells was assessed by OCR analysis after treatment with indicated concentrations of JP1 for 48 hours (*n* = 5). (**C** and **D**) Representative immunohistochemical staining of HIF1α in the B16F10 tumor nodules (**C**). Quantification of HIF1α intensity (**D**) (*n* = 6). (**E** and **F**) Representative immunohistochemical staining of HIF1α in the LLC tumor nodules (**E**). Quantification of HIF1α intensity (**F**) (*n* = 6). Scale bars: 100 μm (**C** and **E**). The solid boxes are further magnified by 8.6×. (**G** and **H**) HIF1α was assessed by Western blot after treatment with indicated concentrations of JP1 for 48 hours in B16F10 (**G**) and LLC (**H**) cells (*n* = 3). (**I** and **J**) HIF1α protein extracted from B16F10 (**I**) and LLC (**J**) tumor nodules was assessed by Western blot; quantitation of HIF1α protein concentrations is shown (*n* = 6). **P < 0.01; ***P < 0.001 by unpaired, 2-tailed Student’s *t* test (**D**, **F**, **I**, and **J**) or ordinary 1-way ANOVA (**G** and **H**).

Hypoxia is the inevitable outcome of tumor progression and has been reported to stimulate IL-8 secretion (31). JWA’s core functional sequence was designed as the antitumor peptide JP1, which improves the tumor microenvironment hypoxia by promoting aerobic respiration and inhibits pancreatic cancer metastasis (29). Analogously, to verify whether JP1 can exert similar functions as JWA to promote tumor cell oxidative phosphorylation, we performed oxygen consumption rate (OCR) analysis. As shown in Figure 4, A and B, JP1 effectively enhanced the oxidative phosphorylation of B16F10 and Lewis lung carcinoma (LLC) cells. HIF1α staining of tumor samples from the melanoma and lung cancer models showed that JP1 significantly improved the intratumor microenvironment hypoxia (Figure 4, C–F). Next, Western blot analysis revealed that HIF1α, HIF2α, and HIF3α prominently decreased with increasing JP1 concentration in B16F10 and LLC cells (Figure 4, G and H, and Supplemental Figure 5, A and B). This finding was subsequently confirmed in the tumor samples from mice (Figure 4, I and J, and Supplemental Figure 5, C and D). Additionally, we separated nuclear and cytoplasmic proteins through a nucleocytoplasmic separation experiment. Western blot analysis showed that JP1 inhibits HIF1α expression in the nucleus and cytoplasm (Supplemental Figure 5, E and F). The qPCR analysis further validated the effect of JP1 on HIF1α in vitro and in vivo (Supplemental Figure 5, G–J). These results indicated that JP1 effectively promotes tumor oxidative phosphorylation and improves tumor microenvironment hypoxia.

### Hypoxia induces tumor cells to secrete IL-8.

**Figure 5:**
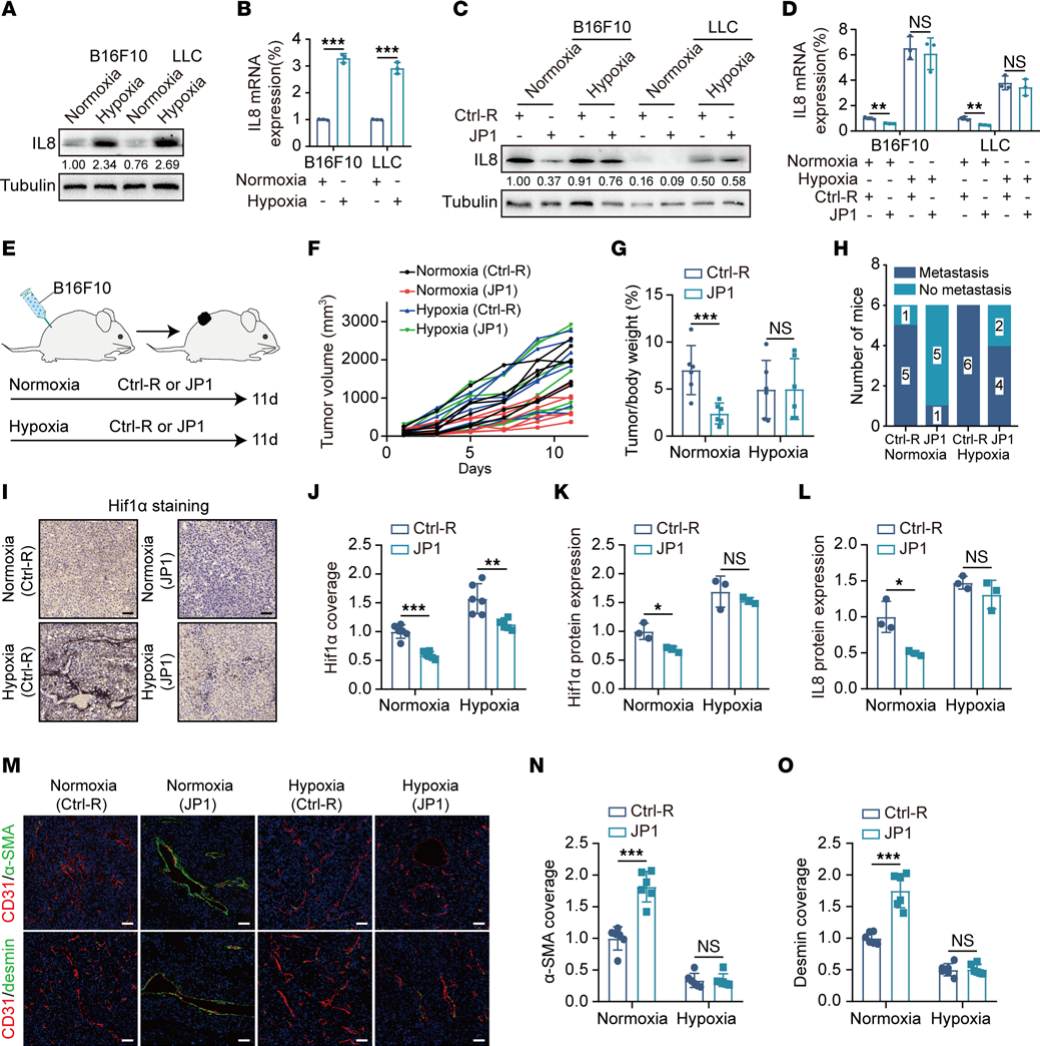
Hypoxia induces tumor cells to secrete IL-8. (**A** and **B**) Western blot (**A**) and qPCR (**B**) analysis of IL-8 in B16F10 and LLC cells after treatment with normoxia or hypoxia for 24 hours (*n* = 3). (**C** and **D**) Western blot (**C**) and qPCR (**D**) analysis of IL-8 in B16F10 and LLC cells after treatment with Ctrl-R or JP1 (50 μM) for 24 hours in normoxic or hypoxic condition (*n* = 3). (**E**) Schematic representation of the B16F10 cell allografts for Ctrl-R (50 mg/kg/day) or JP1 (50 mg/kg/day) treatment in the normoxic or hypoxic condition (*n* = 6). (**F** and **G**) The tumor growth curves and tumor/body weight (%) in indicated groups. (**H**) Quantitation of the mice that developed lung metastases after surgical removal of the primary B16F10 tumors with indicated treatments (*n* = 6). (**I** and **J**) Representative immunohistochemical staining of HIF1α in the B16F10 tumor nodules in indicated groups (**I**) and quantification of HIF1α intensity (**J**) (*n* = 6). Scale bars: 50 μm. (**K** and **L**) Quantitation of HIF1α (**K**) and IL-8 (**L**) protein extracted from B16F10 tumor nodules in indicated groups by Western blot (*n* = 3). (**M**–**O**) Representative fluorescence images of α-SMA (green), desmin (green), CD31 (red), and DAPI nuclear staining in indicated groups (**M**); quantitation of α-SMA (**N**) and desmin (**O**) coverage is shown (*n* = 6). Scale bars: 50 μm. *P < 0.05, **P < 0.01, ***P < 0.001 by ordinary 1-way ANOVA (**B**, **D**, **G**, **J**–**L**, **N**, and **O**).

To determine the effect of tumor microenvironment hypoxia on IL-8 expression, we constructed normoxia and hypoxia models with B16F10 and LLC cells and found that the expression of IL-8 increased under hypoxic conditions (Figure 5, A and B). Moreover, JP1 significantly inhibited the expression of IL-8 under normoxic conditions, while under hypoxic conditions, the regulatory effect of JP1 on IL-8 was weakened (Figure 5, C and D). To further test this hypothesis, we created a tumor-bearing model of melanoma under normoxic and hypoxic rearing environments (Figure 5E). The results indicated that, compared with the significant inhibitory effect of JP1 on tumor growth under normoxic conditions, JP1 had no significant inhibitory effect under hypoxic conditions (Figure 5, F and G, and Supplemental Figure 6A). Subsequently, we conducted a melanoma active metastasis model and found that under a normoxic rearing environment, 5 out of 6 mice in the Ctrl-R group showed lung metastasis, whereas only 1 out of 6 mice had lung metastasis in the JP1 group. Under a hypoxic rearing environment, all 6 mice showed lung metastasis in the Ctrl-R group, and 4 out of 6 mice were observed with lung metastasis in the JP1 group (Figure 5H and Supplemental Figure 6B). HIF1α staining of mouse tumor samples showed that JP1 significantly improved the intratumor microenvironment hypoxia under normoxic conditions, while under hypoxic conditions, intratumoral hypoxia was more severe and JP1 could not reverse the hypoxic state of the intratumor microenvironment (Figure 5, I and J). This result was verified by Western blot analysis (Figure 5K and Supplemental Figure 6C). Additionally, the IL-8 expression of tumor samples increased significantly under hypoxic conditions (Figure 5L and Supplemental Figure 6C). It can be concluded from Figure 5, M–O that the vascular normalization index under hypoxic conditions was lower than the value under normoxia, regardless of JP1 treatment. These results suggested that JP1 improves the tumor microenvironment hypoxia by promoting oxidative phosphorylation of tumor cells, thereby reducing IL-8 secretion.

In terms of mechanism, we referred to the molecular regulation of aerobic respiration by the JWA gene in pancreatic cancer cells, and verified the expression of related molecular proteins in B16F10 and LLC cells. As expected, we confirmed that JP1 regulates mitochondrial metabolic reprogramming of B16F10 and LLC cells through JP1-mediated AMPK/FOXO3a/UQCRC2 signaling, thereby improving the hypoxic state of the tumor microenvironment (Supplemental Figure 6D).

### JP1 promotes intratumoral delivery of PTX and enhances the antitumor effect.

**Figure 6:**
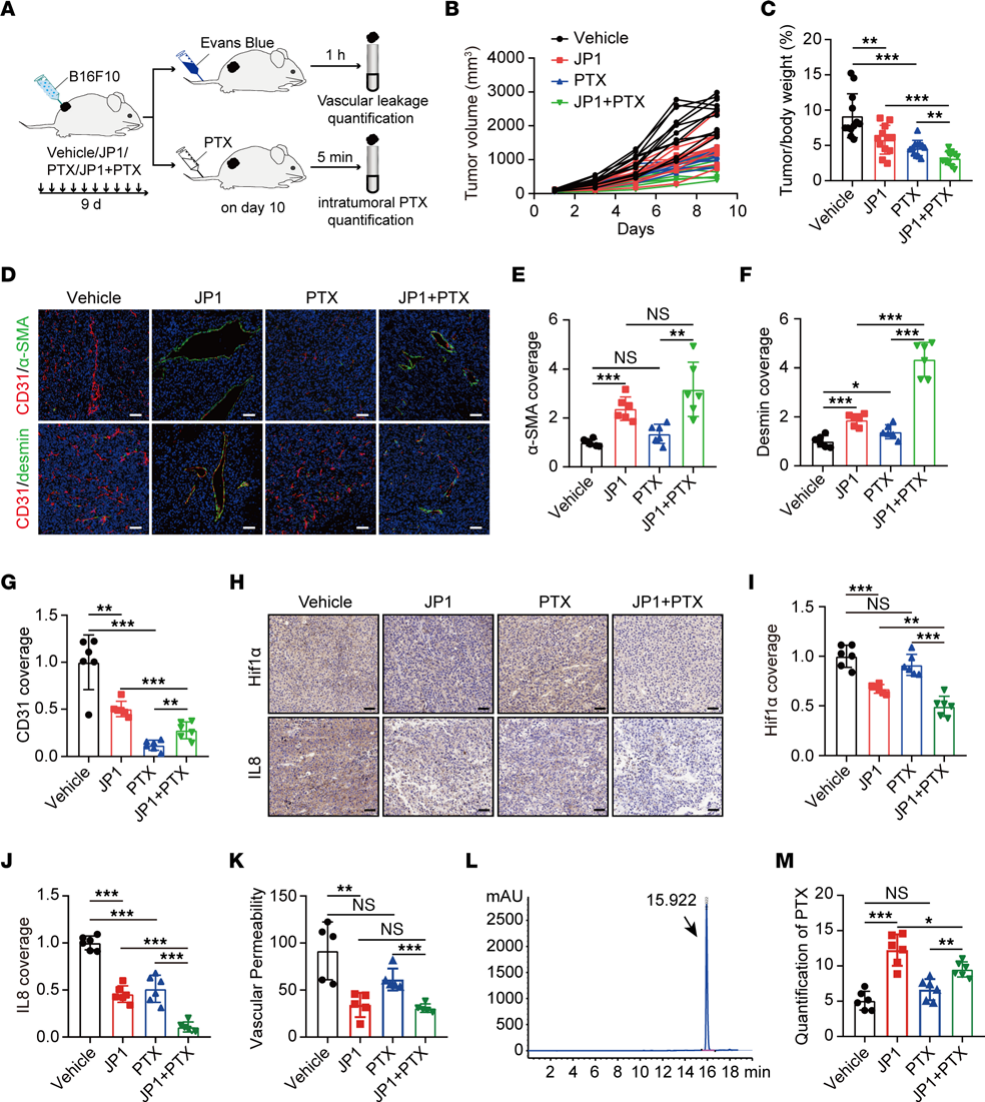
JP1 promotes intratumoral delivery of PTX and enhances the antitumor effect. (**A**) Schematic representation of the B16F10 cell allograft model. Both JP1 and PTX alone or in combination were administered by intraperitoneal injection in mice (*n* = 12). On day 10, six mice were injected with Evans blue to perform vessel permeability assays; 6 mice were injected with PTX to quantify intratumoral PTX concentration. (**B** and **C**) The tumor growth curves and tumor/body weight (%) in indicated groups. (**D**–**G**) Representative fluorescence images of α-SMA (green), desmin (green), CD31 (red), and DAPI nuclear staining in indicated groups (**D**); quantitation of α-SMA (**E**), desmin (**F**), and CD31 (**G**) coverage are shown (*n* = 6). Scale bar: 50 μm. (**H**–**J**) Representative immunohistochemical staining of HIF1α and IL-8 in the B16F10 tumor nodules in indicated groups (**H**). Quantification of HIF1α (**I**) and IL-8 (**J**) intensity (*n* = 6). Scale bars: 50 μm. (**K**) Quantitation of vascular permeability after solvent, JP1, PTX, and JP1 combined with PTX treatment (*n* = 5). (**L**) PTX standard solution exhibits a peak with a retention time of 15.922 minutes by HPLC analysis. (**M**) Quantitation of intratumoral PTX concentrations in indicated groups (*n* = 6). *P < 0.05; **P < 0.01; ***P < 0.001 by ordinary 1-way ANOVA (**C**, **E**–**G**, **I**–**K**, and **M**).

Tumor vascular normalization inhibits tumor metastasis by reducing the invasion of tumor cells into the vasculature (15, 16). Also, the normalized tumor vasculature promotes the delivery of drugs into the intratumoral space and enhances the antitumor effect (32, 33). To evaluate the effect of JP1 in promoting tumor vascular normalization for drug delivery into the intratumoral space, we treated the melanoma-bearing model with JP1 and PTX (Figure 6A). The results showed that JP1 or PTX treatment alone inhibited tumor growth by 37% or 49.7%, respectively, while the inhibitory effect of the combined treatment was 65.4%, suggesting that JP1 combined with PTX has a better antitumor effect (Figure 6, B and C). Tumor vessel morphology validated that, compared with the effect of JP1 in promoting tumor vascular normalization, PTX promoted vascular normalization by decreasing the total number of vessels (Figure 6, D–G). In addition, we performed hypoxia analysis for tumor tissue and found that although PTX promotes tumor vascular normalization, its inhibitory effect on vessels could not improve the hypoxic state, while the combined treatment of JP1 and PTX significantly improved the hypoxic state of the tumor microenvironment (Figure 6, H and I). We subsequently confirmed that, compared with JP1 or PTX treatment alone, JP1 combined with PTX significantly inhibited the expression of IL-8 (Figure 6, H and J). This observation was further validated by vessel permeability assays (Figure 6K). To obtain further evidence that JP1 promotes drug delivery into the intratumoral space, we determined the content of PTX in the tumor by HPLC and found that compared with JP1 treatment, the delivery of PTX into the intratumoral space was significantly lower after 10 days of PTX treatment, while the combined treatment of JP1 and PTX significantly increased the delivery of PTX into the tumor (Figure 6, L and M, and Supplemental Figure 7). Taken together, these results demonstrate that PTX inhibits metastasis initiation by promoting tumor vascular normalization, but its inhibitory effect on vessels reduces the delivery of drugs into the intratumoral space, while JP1 combined with PTX has both functions.

## Discussion

Uncontrollable metastasis is the major contributor to tumor-related death (3). Tumor cells with enhanced invasiveness and vasculature with increased permeability promote metastasis (15, 34). Microenvironmental hypoxia resulting from tumor overgrowth is a prominent factor in strengthening tumor cell invasion and vascular permeability (35, 36). In turn, the disordered vasculature reduces intratumoral perfusion and exacerbates hypoxia, leading to the formation of a malignant feedback loop consisting of hypoxia, vascular disorder, and insufficient perfusion in the tumor microenvironment (37). All of these aberrances are sufficient for tumor cells breaking through growth inhibition and initiating aggressive metastasis in some organs. Therefore, improving the tumor microenvironment hypoxia may effectively inhibit metastasis. In this study, we confirmed that JP1 regulates the mitochondrial metabolic reprogramming of B16F10 and LLC cells through JP1-mediated AMPK/FOXO3a/UQCRC2 signaling, which improves the tumor microenvironment hypoxia. The resulting oxygen-rich tumor microenvironment inhibits the secretion of IL-8 by tumor cells, thereby promoting tumor vascular normalization and inhibiting tumor metastasis. In turn, the normalized vasculature increases intratumoral perfusion, so that the tumor microenvironment regains a benign feedback loop that includes tumor vascular normalization, sufficient perfusion, and an oxygen-rich tumor microenvironment.

Anti–tumor angiogenesis drugs and chemotherapeutic drugs can temporarily promote the normalization of tumor vasculature (38, 39). It has been reported that apatinib, a specific VEGFR-2 inhibitor, temporarily promotes tumor vascular normalization by blocking the transmission of the VEGF/VEGFR-2 signaling pathway and improves pemetrexed therapy. However, the time window of vascular normalization only occurs within 3–7 days after apatinib therapy (40). Furthermore, the antiangiogenesis effect of chemotherapeutic drugs reduces blood perfusion, resulting in ischemia, hypoxia, and acidosis inside the tumor (41, 42). The poor tumor microenvironment accelerates the malignant cloning of tumor cells, which promotes metastasis to distant organs (8). Meanwhile, the poor tumor microenvironment also reduces the chemotherapeutic drug transport in the tumor, thereby inhibiting the curative effect of the chemotherapy (43). This is the main cause of drug resistance and recurrence of tumors after chemotherapy. In the present study, we found that PTX is available to promote tumor vascular normalization but significantly inhibits intratumoral vasculature, which results in decreased intratumoral perfusion and drug delivery. However, JP1 promotes tumor vascular normalization without extensively inhibiting intratumoral vasculature. This is effective for oxygen and drugs to be transported into the tumor and to inhibit ischemia, hypoxia, and acidosis within the tumor. Furthermore, the combined treatment of JP1 and PTX maintains a certain density of vessels in the tumor, promotes tumor vascular normalization, increases the delivery of oxygen and drugs, and enhances the antitumor effect.

Chemotherapeutic drugs are curative therapies for many malignancies and prolong the survival of patients with tumors (44, 45). However, the biophysical effects of chemotherapeutic drugs are not specific to tumor cells and produce multiorgan toxic effects, which limits their clinical application (46). Endowing chemotherapeutic drugs with targeting to tumor cells may significantly reduce toxicity as well as enhance antitumor effects (47, 48). Peptide-drug conjugates (PDCs) are composed of a homing peptide, linker, and cytotoxic payload (49, 50). The homing peptide drives enrichment of the cytotoxic payload in tumor cells by targeting the receptors that are overexpressed on tumor cells. Previous studies have shown that JP1 targeted integrin αvβ3 receptors on tumor cells and ^18^F-labeled JP1 was detected by micro-PET assay to demonstrate tumor targeting in vivo. In combining JP1 with chemotherapeutic drugs into PDCs, chemotherapeutic drugs may achieve the targeting effect on tumor cells through JP1-targeted integrin αvβ3 receptors, thereby reducing the toxic effects on normal organs. In addition, the antitumor effect of JP1 synergizes with chemotherapeutic drugs to inhibit tumors.

**Figure 7:**
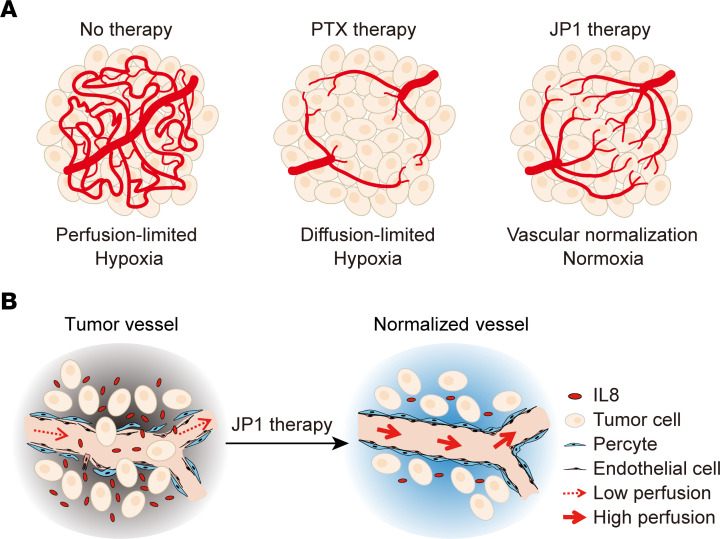
The mechanism of JP1 in inhibiting metastasis initiation. (**A**) Graphical illustration of the tumor microenvironment state after solvent, PTX, and JP1 therapy. (**B**) The working model of JP1 in promoting tumor vascular normalization, thereby inhibiting metastasis initiation.

In this study, we found that the microenvironmental vessels in untreated tumors were disordered and leaky, leading to perfusion-limited hypoxia. PTX promoted tumor vascular normalization, but its inhibitory effect on vessels gave rise to diffusion-limited oxygen and drugs, and eventually developed into a hypoxic tumor microenvironment, while JP1 promoted tumor vascular normalization without extensively inhibiting intratumoral vessels, leading to a mature and stable tumor microenvironment (Figure 7A). Specifically, JP1 regulated the mitochondrial metabolic reprogramming through AMPK/FOXO3a/UQCRC2 signaling, which improves the tumor microenvironment hypoxia. The oxygen-rich tumor microenvironment inhibited the secretion of IL-8 by tumor cells, and then promoted tumor vascular normalization. The normalized vasculature inhibited tumor metastasis and promoted the delivery of PTX into the tumor (Figure 7B).

## Methods

### Cell lines and cell culture.

Both the mouse melanoma B16F10 and lung cancer LLC cells were purchased from ATCC. IL8^KO^ B16F10 cells were generated using *Il8*-specific CRISPR/Cas9 plasmids (synthesized by Corues Biotechnology). Sequencing analysis and Western blotting confirmed the complete gene knockout. GFP-B16F10 cells were generated using GFP-specific plasmids (synthesized by Shanghai Genechem Co., Ltd). All the cell lines were maintained in DMEM supplemented with 100 μg/mL streptomycin, 100 U/mL penicillin, and 10% fetal bovine serum (FBS) in an incubator with 5% CO_2_ at 37°C.

### Mouse allograft assays.

The experimental mice were purchased from SLAC Laboratory Animal Center and maintained in the Animal Core Facility of Nanjing Medical University. In the melanoma growth model, B16F10, IL8^WT^ B16F10, IL8^KO^ B16F10, and GFP-B16F10 cells (5 × 10^5^) were subcutaneously injected into C57BL/6 male mice. In the lung cancer growth model, LLC cells (5 × 10^6^) were subcutaneously injected into C57BL/6 male mice. When the tumor volume reached 100 mm^3^, the mice were randomly grouped, and the body weights and tumor size were measured every other day. At the end of the experiment, the mice were humanely sacrificed and the tumors were measured and recorded. We used an oxygen concentration controller (Shanghai Yuyan Instruments Co., Ltd), which creates an environment containing 8% oxygen, and the mice stayed 6 hours/day to build a hypoxia model. When the mice are kept in the anoxic chamber, the hypoxia of tumor microenvironment becomes an irreversible process. Melanoma/lung cancer active metastasis models were utilized under a previously described general protocol (30).

### CTC analysis and sorting.

The melanoma-bearing model was constructed using GFP-labeled B16F10 cells. When the tumor volume reached 2000 mm^3^, 500 μL of blood per mouse was taken and the red blood cells lysed (Red Blood Cell Lysis Buffer, Fcmacs Biotechnology Company). FACS analysis was performed to detect CTCs per 1.5 million cells using an LSRII Flow Cytometer (BD Biosciences), and the data were analyzed using FlowJo software (Tree Star). For sorting, FACSAria III instruments (BD Biosciences) were used.

### Cell proliferation, migration, and invasion assays.

For proliferation assay, blood from melanoma-bearing mice was taken and red blood cells lysed, and the cells were cultured in DMEM for 21 days. The FACS-isolated CTCs were used for migration and invasion assays. For migration assay, CTCs were inoculated in the serum-free medium in the upper chamber of a Transwell filter (Corning), and DMEM containing 10% FBS was added to the lower chamber. After 12 hours, cells were fixed with methanol for 1 hour, stained with crystal violet (Beyotime) for 30 minutes, and counted. For invasion assays, a layer of Matrigel (BD Biosciences) was added to the membrane of the lower chamber.

### Immunohistochemistry and immunofluorescent staining.

The tissue specimens were immersed in formalin for 24 hours, and then embedded in paraffin and sectioned (paraffin microtome, HM340E, Thermo FIsher Scientific). After deparaffinization and antigen retrieval, the slides were blocked with 10% normal goat serum for 30 minutes at room temperature, and incubated with anti-HIF1α (36169T, Cell Signaling Technology) antibody solution for 24 hours at 4°C with gentle shaking. The images were scanned with a pathology section scanner (Pannoramic MIDI) after DAB staining. For dual immunofluorescent staining, mouse anti-CD31 (GB12063, Servicebio) antibody was incubated with rabbit anti–α-SMA (GB111364, Servicebio), anti–claudin 5 (GB11290, Servicebio), and anti-desmin (GB11081, Servicebio) antibody solution for 24 hours at 4°C with gentle shaking. The images were scanned after incubation with mouse-derived (red) and rabbit-derived (green) fluorescent secondary antibodies. The staining intensity was analyzed with ImageJ (NIH).

### Vessel permeability assays.

The melanoma tumor–bearing mice were injected with 50 mg/kg Evans blue solution through the tail vein. After 1 hour, the blood was collected, centrifuged to obtain the supernatant, and diluted 1:100 with formamide. Evans blue was dissolved in formamide (0, 125, 250, 500, 1000, 5000, 10,000, 25,000, and 50,000 ng/mL) to generate a standard curve. The supernatant absorbance was measured at 620 nm to calculate the concentration of Evans blue in the blood. After the blood was taken, mouse hearts were quickly exposed, and the heart was perfused with 10 mL 0.9% saline solution to wash the Evans blue into the receiving vessel. Tumor tissue (200 mg) was placed in a 60°C oven for 5 hours, at which point 200 μL formamide was added, followed by 60°C incubation for 18 hours. The supernatant was centrifuged and the absorbance measured at 620 nm to calculate the Evans blue content in the tumor tissue. Vessel permeability was calculated using the following formula: vessel permeability (μL/[g × h]) = (Evans blue [μg]/tumor dry weight [g])/(Evans blue concentration in blood [μg/μL] × circulation time [h]).

### Angiogenesis array.

Angiogenesis Array GS1000 (GSH-ANG-1000-1, Ray Biotech Inc.) was used according to the manufacturer’s instructions. In brief, the glass slides were allowed to equilibrate to room temperature inside a sealed plastic bag for 20–30 minutes, after which the slides were uncovered and allowed to air dry for another 1–2 hours. The standards or sample were collected and incubated with the arrays for 24 hours at 4°C with gentle shaking. The chemiluminescent signals were captured using an InnoScan 300 Microarray Scanner (Innopsys). Data extraction was done using the microarray analysis software ScanArray Express (PerkinElmer).

### qPCR and Western blot analysis.

Total RNA from cells and tissues was extracted with TRIzol (10296010, Thermo Fisher Scientific), and reverse transcription was performed with a reverse transcription kit (R323-01, Vazyme). qPCR was performed with SYBR (TSE202, Vazyme) in an Applied Biosystems QuantStudio 5 Real-Time PCR system. Finally, the relative expression was calculated using GAPDH as a standard. The primers for qPCR are listed in Supplemental Table 1. Western blot analysis was conducted as reported previously. Briefly, cells and tissue samples were lysed in cell lysis buffer and tissue protein extraction reagent (78510, Thermo Fisher Scientific), respectively. A total of 40 μg protein was processed for subsequent analysis. Antibodies against HIF1α (36169T, Cell Signaling Technology), IL-8 (ab106350, Abcam), p-AMPK (2535T, Cell Signaling Technology), AMPK (ab32047, Abcam), FOXO3A (ab53287, Abcam), UQCRC2 (ab203832, Abcam), and tubulin (AF0001, Beyotime) were used.

### ELISA.

B16F10 cell supernatants were collected after Ctrl-R or JP1 (50 μM for 48 hours) treatment. ELISAs were used to detect the expression of IL-8, IL-6, IL-1β, and HB-EGF according to the manufacturer’s protocol (Baolai Biotechnology Company).

### Mitochondrial OCR analysis.

OCR analysis of B16F10 and LLC cells were performed using a Seahorse XFp Analyzer (Seahorse XF96, Agilent). B16F10 cells (6000) and LLC cells (10,000) were seeded into Seahorse plates in growth medium for 24 hours. The mitochondrial OCR was measured in XF base medium containing 1 mM sodium pyruvate, 2 mM L-glutamine, and 10 mM glucose following sequential additions of oligomycin (1 μM) and FCCP (B16F10: 1.5 μM; LLC: 1 μM).

### HPLC assays.

PTX was purchased from Selleck (NSC 125973), HPLC (column: Agilent Zorbax Exlipse Plus C18, 100 × 4.6 mm, 3.5 μm particle size; flow rate: 1 mL/min; wavelength: 254 nm; mobile phase [0.1% TFA in CH_3_OH/0.1% TFA in H_2_O] 0 min: 10:90; 0–15 min: 10:90 to 100:0; 15–20 min: 100:0) was used to determine the peak retention time of PTX. The melanoma allograft model was conducted as described in *Mouse allograft assays* above. The experimental groups were as follows: solvent (inject every day); JP1 (50 mg/kg, inject every day); PTX (10 mg/kg, inject once every 3 days); and JP1 + PTX (JP1: 50 mg/kg, inject every day; PTX: 10 mg/kg, inject once every 3 days). On day 10, melanoma tumor–bearing mice were injected with 20 mg/kg PTX solution through the tail vein. After 5 minutes, 200 mg of the tumor was taken and dissolved in 1 mL of methanol. PTX was dissolved in methanol (200, 100, 50, 25, 12.5, 6.25, 3.125, and 1.56 μg/mL) to generate a standard curve. The standard and samples were measured by HPLC to calculate the concentration of PTX in the tumor.

### Statistics.

All data are expressed as mean ± SEM of individual samples, and were analyzed using GraphPad Prism 8 and SPSS 20 software (IBM). Statistical significance between experimental groups was determined using an unpaired, 2-tailed Student’s *t* test or 1-way ANOVA. A *P* value of less than 0.05 was considered statistically significant: *P < 0.05; **P < 0.01; ***P < 0.001; NS, no significance.

### Study approval.

All care and treatment of experimental animals were approved by the Institutional Animal Care and Use Committee of Nanjing Medical University (Nanjing, China).

## Author contributions

YS and JZ designed and supervised the project. JC, ZC, LZ, KD, HJ, and AL contributed to design and performed functional experiments in vivo and in vitro. DC contributed to HPLC assays. ZX contributed to vessel permeability assays. JC, ZC, JZ, and YS prepared the manuscript.

## Supplementary Material

Supplemental data

Supplemental table 2

## Version 1 05/16/2023

In-Press Preview

## Version 2 06/22/2023

Electronic publication
