# Supplementary material for: JP1 normalizes tumor vasculature to suppress metastasis and facilitate drug delivery by inhibiting IL-8
Source: JCI Insight. 2023 Jun 22;8(12):e161675. doi: 10.1172/jci.insight.161675 (PMC10371244; doi:10.1172/jci.insight.161675)
Supplement: Supplemental data [file jciinsight-8-161675-s213.pdf]

JP1 normalizes tumor vessels to suppress metastasis and facilitate drug delivery by inhibiting IL8

Jiahua Cui[1], Zhen Che[2, 3], Lu Zou[2, 3], Dongyin Chen[4], Zhan Xie[5], Kun Ding[2, 3], Huning Jiang[1], Aiping Li[2, 3], Jianwei Zhou[2, 3*], Yongqian Shu[1, 6, 7*]

Supplementary figures

Supplementary Figure 1. JP1 inhibits melanoma and lung cancer metastasis initiation

Supplementary Figure 2. JP1 inhibits tumor cells into the vasculature

Supplementary Figure 3. JP1 inhibits the expression of IL8 in vitro and vivo

Supplementary Figure 4. JP1 reduces metastasis initiation by inhibiting IL8

Supplementary Figure 5. JP1 inhibits the expression of HIF1α in vitro and vivo

Supplementary Figure 6. JP1 inhibits IL8 by regulating the mitochondria metabolic reprogramming

Supplementary Figure 7. HPLC analyzes the content of PTX in the tumor

Supplemental Table 1: Primers for qRT-PCR

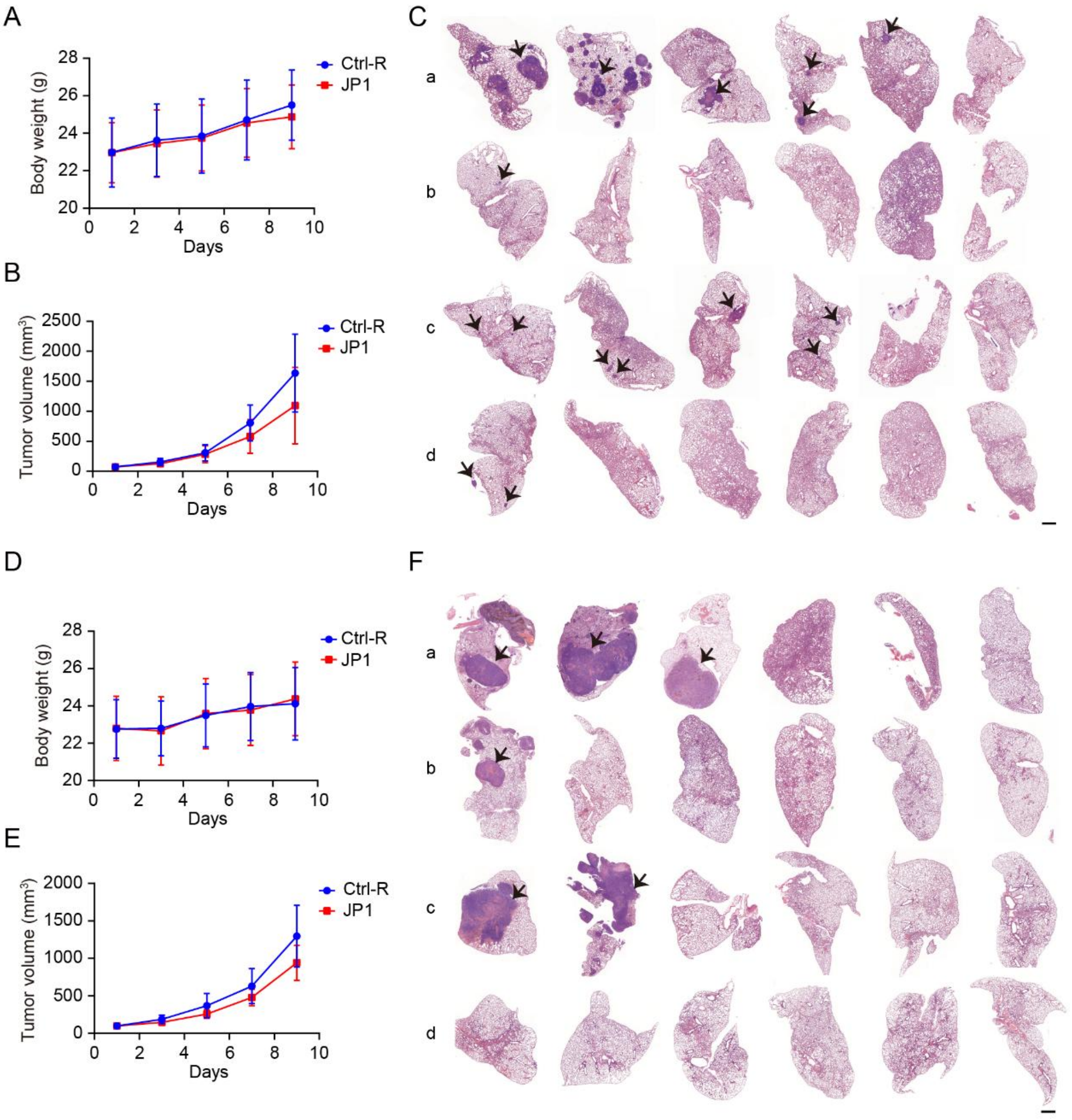

Supplementary Figure 1. JP1 inhibits melanoma and lung cancer metastasis initiation

(A-B) The body weights and tumors volume measurements in B16F10 tumor after Ctrl-R or JP1 treatment. **(C)** Representative lung HE staining images in indicated groups to detect B16F10 tumor metastasis initiation. Scale bar: 500 µm. **(D-E)** The body weights and tumors volume measurements in LLC tumor after Ctrl-R or JP1 treatment. **(F)** Representative lung HE staining images in indicated groups to detect LLC tumor metastasis initiation. Scale bar: 500 µm.

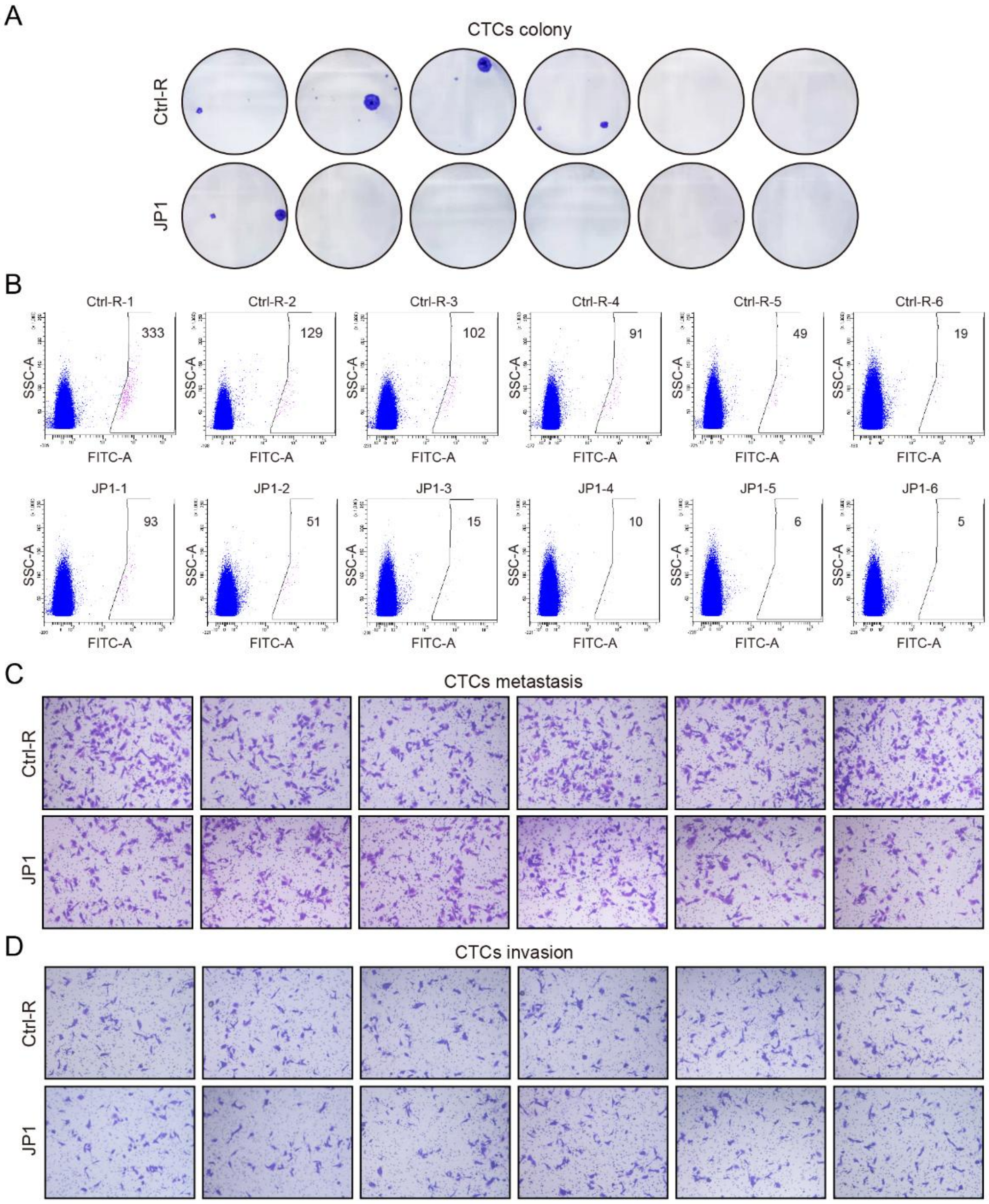

Supplementary Figure 2. JP1 inhibits tumor cells into the vasculature

(A) Representative images of CTCs colony formation after Ctrl-R or JP1 treatment. **(B)** Representative images from CTCs quantitation per 1.5×10^7 hematocytes by FACS analysis after Ctrl-R or JP1 treatment. **(C)** Representative images of CTCs migration after Ctrl-R or JP1 treatment. **(D)** Representative images of CTCs invasion after Ctrl-R or JP1 treatment.

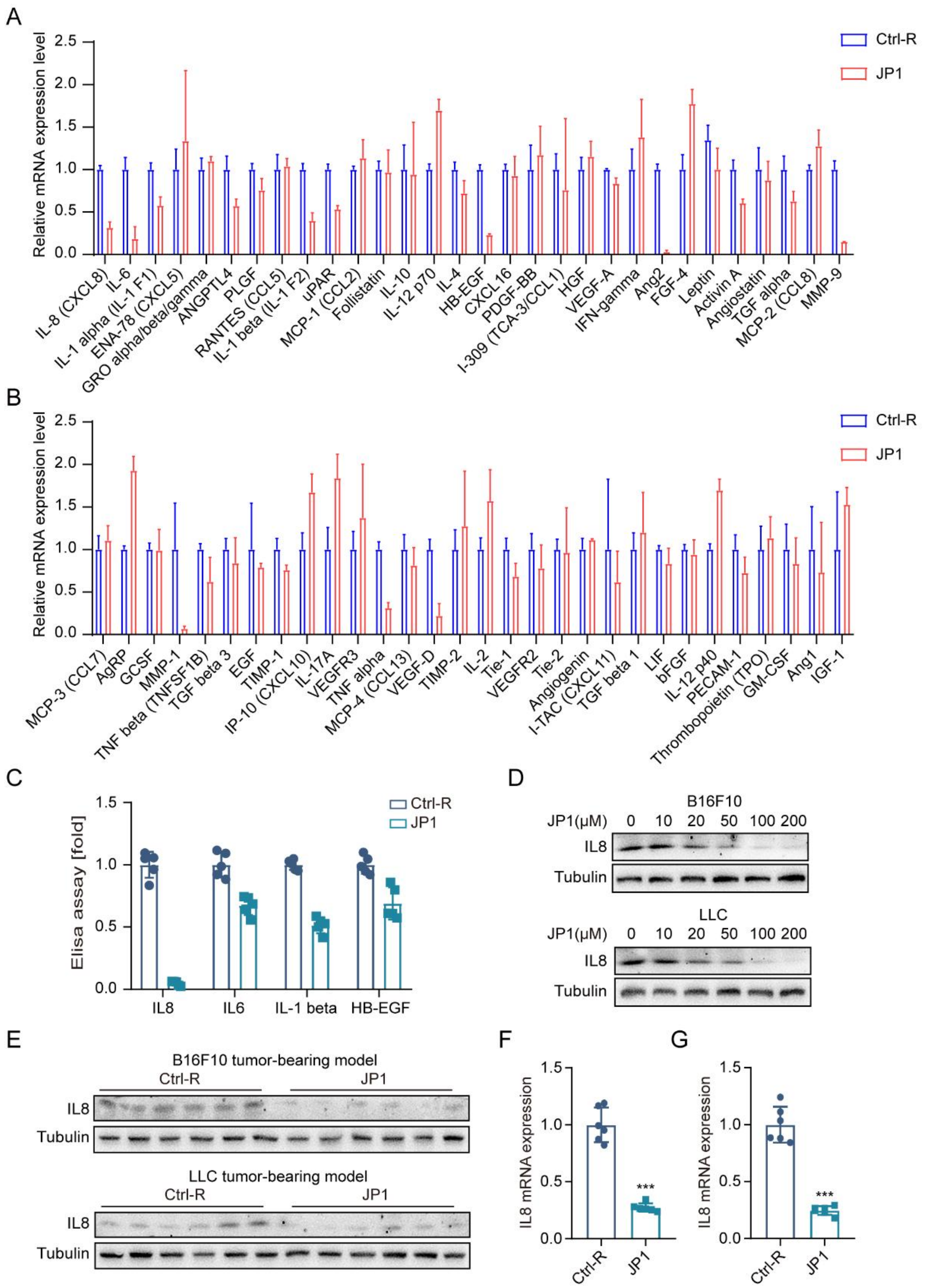

Supplementary Figure 3. JP1 inhibits the expression of IL8 in vitro and vivo

(A-B) Quantitation of 60 genes mRNA after JP1 treatment in B16F10 cells. **(C)** The expression of IL8, IL6, IL-1 beta, HB-EGF in the supernatant of B16f10 cells were detected after JP1 treatment by Elisa analysis. **(D)** Representative Western Blot

analysis was performed on whole-cell lysates from B16F10 and LLC cells after JP1 treatment in indicated concentrations to assess the expression of IL8. **(E)** Representative Western Blot analysis of IL8 protein that extracted from B16F10 and LLC tumor nodules. **(F-G)** The mRNA of IL8 extracted from B16F10 (F) and LLC (G) tumor nodules was assessed by qPCR analysis; quantitation of IL8 mRNA expression were shown. ***P < 0.001 by unpaired two-tailed Student' s t-test (F and G).

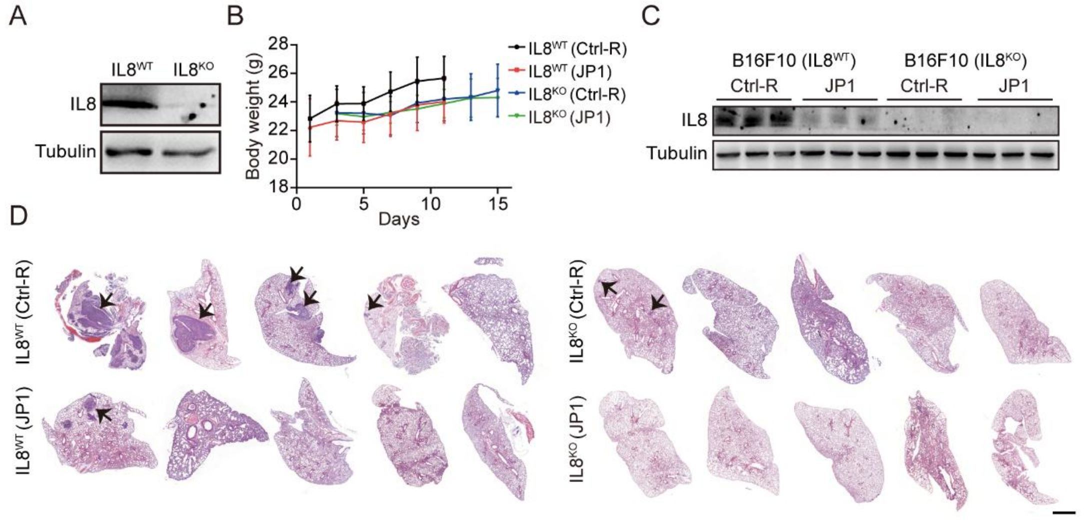

Supplementary Figure 4. JP1 reduces metastasis initiation by inhibiting IL8

(A) Representative Western Blot analysis indicated the efficiency of IL8 knockout. **(B)** The body weights of mice in indicated groups. **(C)** Representative Western Blot analysis of IL8 protein that extracted from $IL8^{WT}$ and $IL8^{KO}$ B16F10 tumor after Ctrl-R or JP1 treatment. **(D)** Representative lung HE staining images in indicated groups to detect B16F10 tumor metastasis initiation. Scale bar: 500 μm.

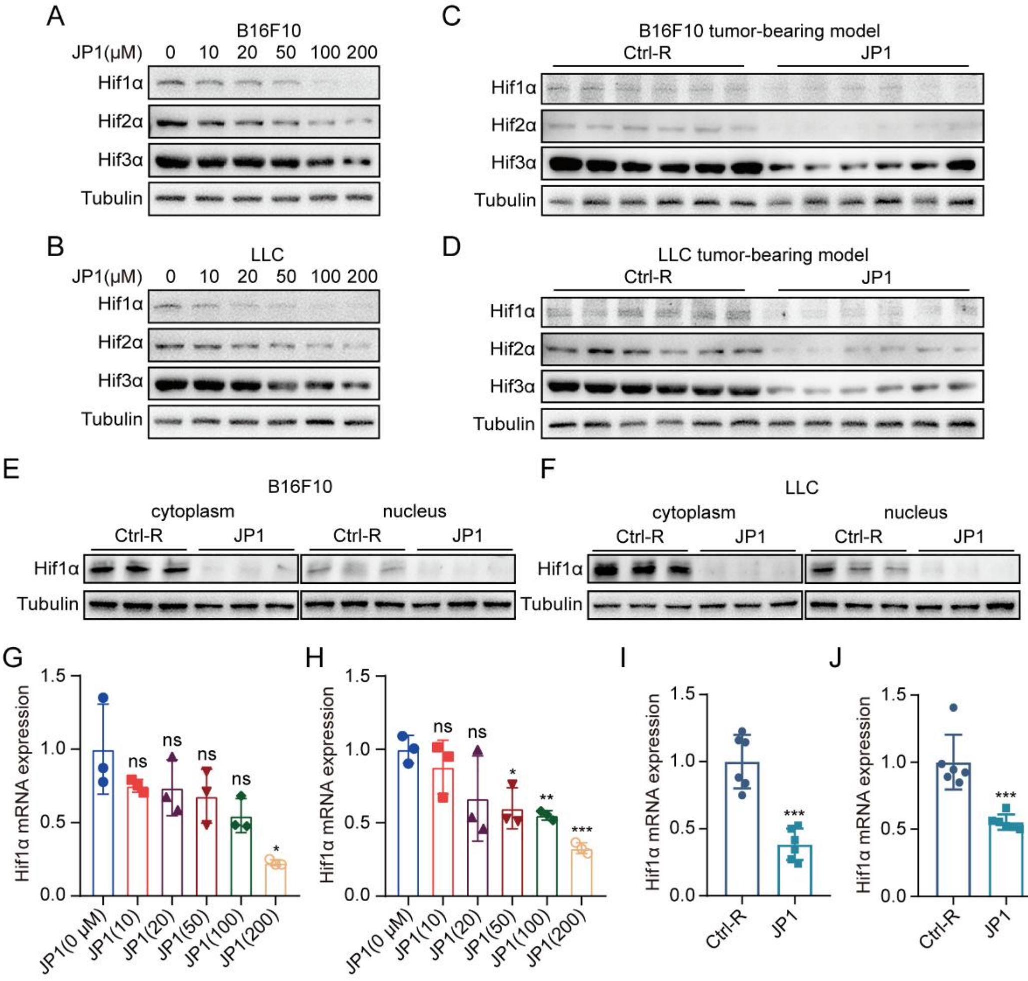

Supplementary Figure 5. JP1 inhibits the expression of HIF1α in vitro and vivo

(A-B) Representative Western Blot analysis was performed on whole-cell lysates from B16F10 (A) and LLC (B) cells after JP1 treatment in indicated concentrations to assess the expression of HIF1α, HIF2α and HIF3α. **(C-D)** Representative Western Blot analysis of HIF1α, HIF2α and HIF3α protein that extracted from B16F10 (C) and LLC (D) tumor nodules. **(E-F)** Representative Western Blot analysis of HIF1α protein that extracted from the cytoplasm/nucleus of B16F10 (E) and LLC (F) cells. **(G-H)** Quantitation of HIF1α mRNA after JP1 treatment in indicated concentrations in B16F10 (G) and LLC (H) cells. **(I-J)** The mRNA of HIF1α extracted from B16F10 (I) and LLC (J) tumor nodules was assessed by qPCR analysis; quantitation of HIF1α

mRNA expression were shown in panel. *P value < 0.05, **P < 0.01, and ***P < 0.001 by ordinary one-way ANOVA (G and H) or unpaired two-tailed Student' s t-test (I and J).

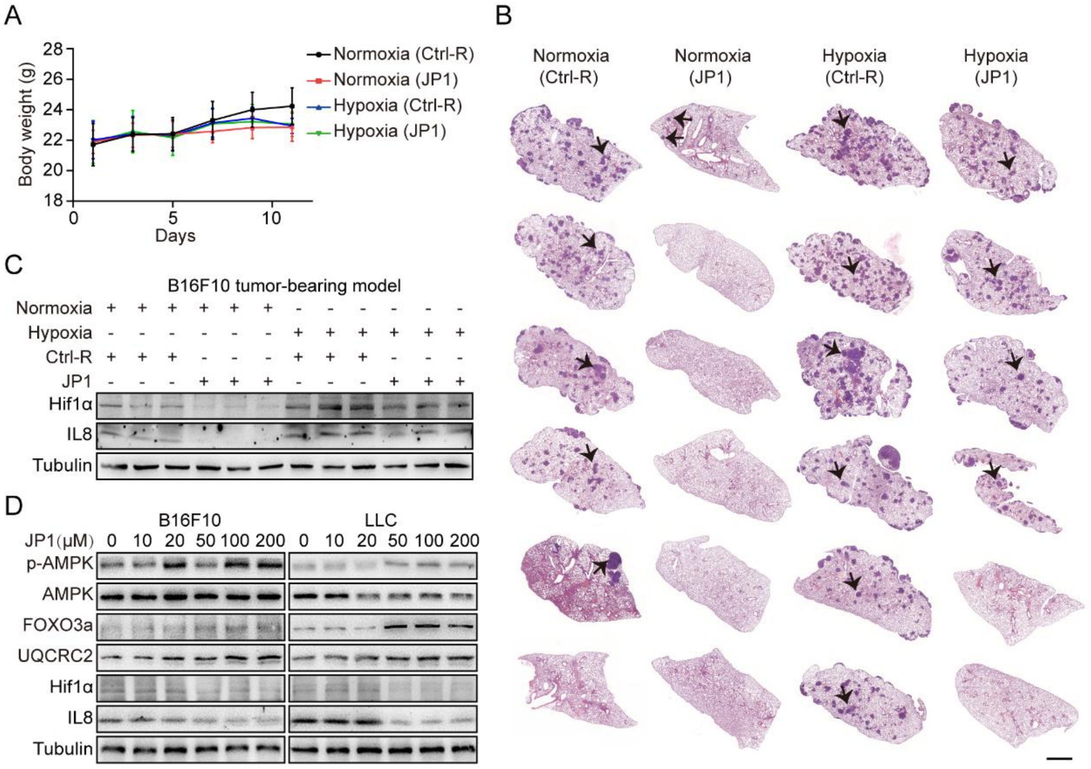

Supplementary Figure 6. JP1 inhibits IL8 by regulating the mitochondria metabolic reprogramming

(A) The body weights of mice in indicated groups. **(B)** Representative lung HE staining images in indicated groups to detect B16F10 tumor metastasis initiation. Scale bar: 500 μm. **(C)** Representative Western Blot analysis of HIF1α and IL8 protein that extracted from B16F10 tumor nodules with Ctrl-R or JP1 treatment under normoxia and hypoxia state. **(D)** Representative Western Blot analysis was performed on whole-cell lysates from B16F10 and LLC cells after JP1 treatment in indicated concentrations to assess the expression of p-AMPK, AMPK, FOXO3a, UQCRC2, HIF1α and IL8.

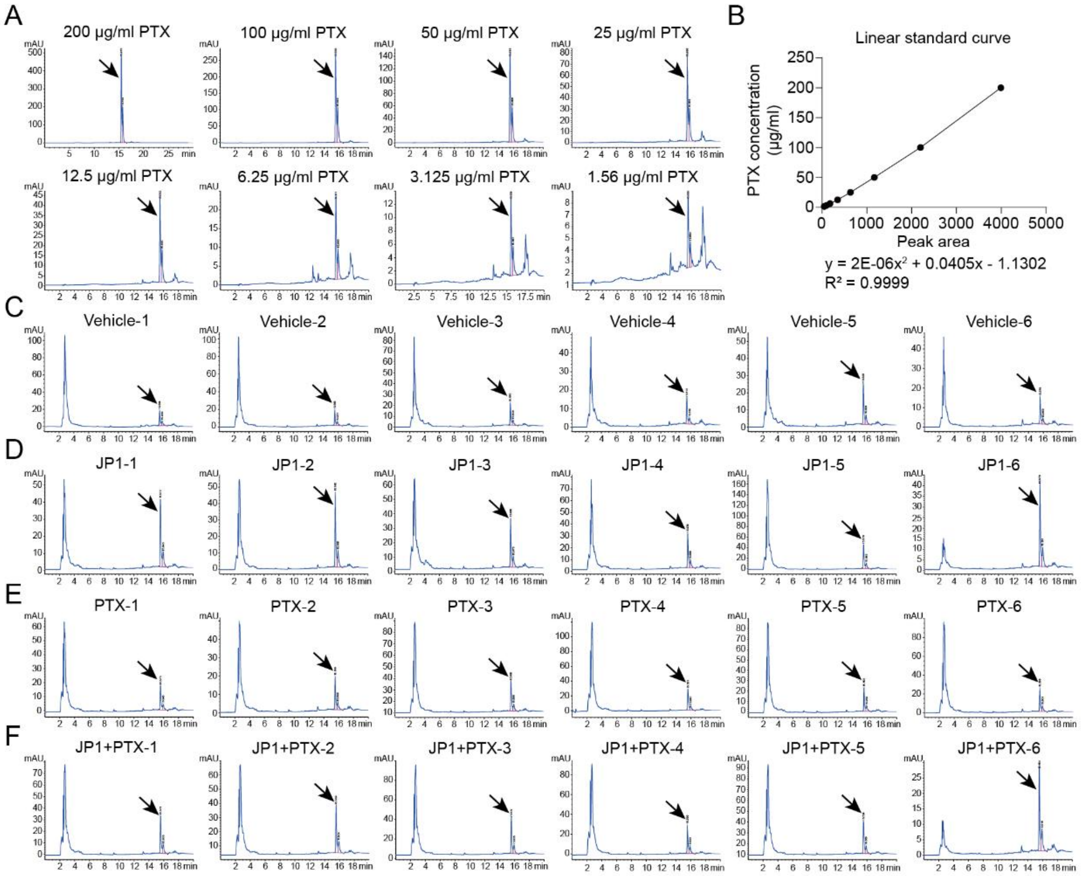

Supplementary Figure 7. HPLC analyzes the content of PTX in the tumor

(A) Representative HPLC assays images of PTX in indicated concentrations. **(B)** The linear standard curve of PTX concentration and peak area. **(C-F)** Representative HPLC assays images of PTX in indicated groups.

Supplemental Table 1: Primers for qRT-PCR

Activin A (Ho-F GTGAGTGCCCGAGCCATATAG

Activin A (Ho-R CATGCGGTAGTGGTTGATGACT

AgRP (Ho-F AGGCCTTGGCAGAGGTACTA

AgRP (Ho-R GCAGGACTCATGCAGCCTTA

Angiopoietin-1 (Ho)-F CCTGATCTTACACGGTGCTGATT

Angiopoietin-1 (Ho)-R GTCCCGCAGTATAGAACATTCCA

Angiopoietin-2 (Ho)-F ATAAGCAGCATCAGCCAACCA

Angiopoietin-2 (Ho)-R CATTCCGTTCAAGTTGGAAGGA

Angiogenin (Ho)-F GGGCGTTTTGTTGTTGGT

Angiogenin (Ho)-R ATCATAGTGCTGGGTCAGGAAG

Angiostatin (Ho)-F CACAGCTGAGAGGGAAATCGTGCG

Angiostatin (Ho)-R TTGCGGTGCACGATGGAGGGGCCGG

ANGPTL4 (Ho)-F GATGGCTCAGTGGACTTCAACC

ANGPTL4 (Ho)-R TGCTATGCACCTTCTCCAGACC

bFGF (Ho)-F TCAAGCAGAAGAGAGAGGAG

bFGF (Ho)-R CCGTAACACATTTAGAAGCC

CXCL16 (Ho)-F ACTACACGACGTTCCAGCTCC

CXCL16 (Ho)-R CTTTGTCCGAGGACAGTGATC

EGF (Ho)-F TGCCCTCAACCTTGGTTTGT

EGF (Ho)-R GGTTGCATTGACCCATCTGC

ENA-78 (CXCL5) (Ho)-F CCTTTTCTAAAGAAAGTCATCCAGA

ENA-78 (CXCL5) (Ho)-R TGGGTTCAGAGACCTCCAGA

FGF-4 (Ho)-F GATGAGTGCACGTTCAAGGA

FGF-4 (Ho)-R GGTTCCCCTTCTTGGTCTTC

Follistatin (Ho)-F GTCGGGATGTTTTCTGTCCAG

Follistatin (Ho)-R TGGCATAAGTGGCATTGTCAC

GCSF (Ho)-F TGCTTAGAGCAAGTGAGGAAGATC

GCSF (Ho)-R GCACACTCACTCACCAGCTTCT

GM-CSF (Ho)-F AAATGTTTGACCTCCAGGAGCCGA

GM-CSF (Ho)-R AGGTGATAATCTGGGTTGCACAGG

GRO alpha/beta/gamma (Ho)-F CCCAAACCGAAGTCATAGCC

GRO alpha/beta/gamma (Ho)-R CAGGAACAGCCACCAGTGAG

HB-EGF (Ho)-F ATCGTGGGGCTTCTCATGTTT

HB-EGF (Ho)-R TTAGTCATGCCCAACTTCACTTT

HGF (Ho)-F CTGGTTCCCCTTCAATAGCA

HGF (Ho)-R CTCCAGGGCTGACATTTGAT

I-309 (TCA-3/CCL1) (Ho)-F TGGATGGAATGACGTAGGGTTGGA

I-309 (TCA-3/CCL1) (Ho)-R TGTCCTGGTTGAGTGGGAAGCAAA

IFN-gamma (Ho)-F TCGGTAACTGACTTGAATGTCCA

IFN-gamma (Ho)-F TCGCTTCCCTGTTTTAGCTGC

IGF-1 (Ho)-F GCTCTTCAGTTCGTGTGTGGA

IGF-1 (Ho)-R GCCTCCTTAGATCACAGCTCC

IL-10 (Ho)-F CTTACTGACTGGCATGAGGATCA

IL-10 (Ho)-R GCAGCTCTAGGAGCATGTGC

IL-12 p40 (Ho)-F ACCATCCAAGTCAAAGAA

IL-12 p40 (Ho)-R TATCAGGAGGAACGAATG

IL-12 p70 (Ho)-F CTGGAGCACTCCCCATTCCTA

IL-12 p70 (Ho)-R GCAGACATTCCCGCCTTTG

IL-17A (Ho)-F CTGTCCCCATCCAGCAAGAG

IL-17A (Ho)-R AGGCCACATGGTGGACAATC

IL-1 alpha (IL-1 F1) (Ho)-F CAACCAGTGCTGAAGGAG

IL-1 alpha (IL-1 F1) (Ho)-R TGCCGTGAGTTTCCCAGAAG

IL-1 beta (IL-1 F2) (Ho)-F AAACAGATGAAGTGCTCCTT

IL-1 beta (IL-1 F2) (Ho)-R TGGAGAACACCACTTGTTGC

IL-2 (Ho)-F TTACATGCCCAAGAAGGCCA

IL-2 (Ho)-R AGCACTTCCTCCAGAGGTTTG

IL-4 (Ho)-F CGGCAACTTTGTCCACGGA

IL-4 (Ho)-R TCTGTTACGGTCAACTCGGTG

IL-6 (Ho)-F CCTTAAAGCTGCGCAGAATG

IL-6 (Ho)-R ATTCAATGAGGAGACTTGCC

IL-8 (CXCL8)-F TCCTGCTTTCTGCAGCTCTC

IL-8 (CXCL8)-R GGGTGGAAAGGTGTGGAATG

IP-10 (CXCL10)-F AGAGGAACCTCCAGTCTCAGC

IP-10 (CXCL10)-R CCTCTGTGTGGTCCATCCTT

I-TAC (CXCL11)-F GCTATAGCCTTGGCTGTGATATTGTG

I-TAC (CXCL11)-R CTGCCACTTTCACTGCTTTTACC

Leptin-F ACCACCCCCAAATTTT

Leptin-R AGATTAGTAGAGAAGGAGGAAGGA

LIF-F GTCTTGGCGGCAGTACACAG

LIF-R CGACTATGCGGTACAGCTCC

MCP-1 (CCL2)-F AAGCTGTGATCTTCAAGACC

MCP-1 (CCL2)-R TGGAATCCTGAACCCACTTC

MCP-2 (CCL8)-F TCATGCTGAAGCTCACACCCTT

MCP-2 (CCL8)-R AGAATTGCCATTGCACAACTCTT

MCP-3 (MARC/CCL7)-F GCCTCTGCAGCACTTCTGTG

MCP-3 (MARC/CCL7)-R CACTTCTGTGTGGGGTCAGC

MCP-4 (CCL13)-F AGCCAGATGCACTCAACGTC

MCP-4 (CCL13)-R TCTCCTTGCCCAGTTTGGTT

MMP-1-F CTGAAGGTGATGAAGCAGCC

MMP-1-R AGTCCAAGAGAATGGCCGAG

MMP-9-F CCTGGAGACCTGAGAACCAATC

MMP-9-R TTCGACTCTCCACGCATCTCT

PDGF-BB-F TGGCAGACTCTGAGAGAGAGA

PDGF-BB-R GCTTGGATGAGGGTGCAGAT

PECAM-1 (CD31)-F ATTGCAGTGGTTATCATCGGAGTG

PECAM-1 (CD31)-R CTGGTTGTTGGAGTTCAGAAGTGG

PLGF-F TTGTCTGCTGGGAACGGCTCGT

PLGF-R CCGGCACACAGTGCAGATTCT

RANTES (CCL5)-F GAGTATTTCTACACCAGTGGCAAG

RANTES (CCL5)-R TCCCGAACCCATTTCTTCTCT

TGF alpha-F GGCGCCCTCGATCTCTTTT

TGF alpha-R TGCACCAACGTACCCAGAAT

TGF beta 1-F CTTCAGCTCCACAGAGAAGAACTGC

TGF beta 1-R CACGATCATGTTGGACAACTGCTCC

TGF beta 3-F GGTTTTCCGCTTCAATGTGT

TGF beta 3-R GCTCGATCCTCTGCTCATTC

Tie-1-F AGGTGTTGTTTCCTGTGCCA

Tie-1-R AGGGATTTTGCCAGGTCCAG

Tie-2-F CCTTGGCTCTGCTGGAATGA

Tie-2-R CACGTTTTGGAAGGCTTGGG

TIMP-1-F GACGGCCTTCTGCAAT T CC

TIMP-1-R GTATAAGGTGGTCTGGTTGACTTCTG

TIMP-2-F CCCCCTCTTCAGCAGTG

TIMP-2-R GCGTGTCCCAGGGCACAATGA

TNF alpha-F CCAGGGACCTCTCTCTAATCAG

TNF alpha-R GGTAGGAGACGGCGATGC

TNF beta (TNFSF1B)-F TACACCTCCTCCTTCTGGGG

TNF beta (TNFSF1B)-R TCCAATGAGGTGAGCAGCAG

Thrombopoietin (TPO)-F TCCTAACTGCAAGGCTAACG

Thrombopoietin (TPO)-R AGGGACCTGGAGGTTTGGTT

uPAR-F GCCCAATCCTGGAGCTTGA

uPAR-R TCCCCTTGCAGCTGTAACACT

VEGF-A-F GCTTGCCATTCCCCACT

VEGF-A-R CCGTCTCTCTCTTCCTCG

VEGFR2-F TTTGGTTCTGTCTTCCAAAGT

VEGFR2-R ATGCTCAGCAGGATGGCAA

VEGFR3-F CCCACGCAGACATCAAGACG

VEGFR3-R TGCAGAACTCCACGATCACC

VEGF-D-F TGGAACAGAAGACCACTCTCATCT

VEGF-D-R GCAACGATCTTCGTCAAACATC

GAPDH-F GCTCTCTGCTCCTCCTGTTC

GAPDH-R ACGACCAAATCCGTTGACTC

HIF1α-F ACGTTCCTTCGATCAGTTGTCACC

HIF1α-R GGCAGTGGTAGTGGTGGCATTAG
